# Conductive Scaffolds for Bone Tissue Engineering: Current State and Future Outlook

**DOI:** 10.3390/jfb13010001

**Published:** 2021-12-21

**Authors:** Damion T. Dixon, Cheryl T. Gomillion

**Affiliations:** 1 School of Environmental, Civil, Agricultural and Mechanical Engineering, University of Georgia, Athens, GA 30602, USA; damion@uga.edu; 2 School of Chemical, Materials and Biomedical Engineering, University of Georgia, Athens, GA 30602, USA

**Keywords:** conductive biomaterials, bone tissue engineering, piezoelectricity, bone regeneration, electrical cell response, bone scaffolds

## Abstract

Bone tissue engineering strategies attempt to regenerate bone tissue lost due to injury or disease. Three-dimensional (3D) scaffolds maintain structural integrity and provide support, while improving tissue regeneration through amplified cellular responses between implanted materials and native tissues. Through this, scaffolds that show great osteoinductive abilities as well as desirable mechanical properties have been studied. Recently, scaffolding for engineered bone-like tissues have evolved with the use of conductive materials for increased scaffold bioactivity. These materials make use of several characteristics that have been shown to be useful in tissue engineering applications and combine them in the hope of improved cellular responses through stimulation (i.e., mechanical or electrical). With the addition of conductive materials, these bioactive synthetic bone substitutes could result in improved regeneration outcomes by reducing current factors limiting the effectiveness of existing scaffolding materials. This review seeks to overview the challenges associated with the current state of bone tissue engineering, the need to produce new grafting substitutes, and the promising future that conductive materials present towards alleviating the issues associated with bone repair and regeneration.

## 1. Introduction

Bone related injuries and disorders are of growing concern due to an ever increasing elderly population (65 years and older), both in the United States (U.S.) and globally [1]. These concerns are particularly due to the negative effects increased age has on bone fracture healing and the elevating incidence associated with increased age [2]. Typical bone related defects resulting from fractures and diseases, like osteoporosis, have been projected to increase substantially with this overall surge in the median age of the population [2,3]. The growing rates of disorder occurrence, the reoccurring problems associated with these disorders, and the subsequent reduction in the structural integrity of remaining native bone have motivated researchers in various fields to look further into the development of new bone tissue replacements. Fractured or broken bones are consistently among some of the most common traumatic injuries sustained by humans, regardless of race, age, or gender. As a result of this trend, bone is the second most frequently transplanted tissue worldwide, with well over two million total operations performed each year; over 500,000 of such operations are in the U.S. alone [4].

Although bone is a thoroughly studied tissue with most of its biological and physical properties being well known, there is still an immense challenge when attempting to replicate these characteristics outside of living organisms. Bone tissue is a specialized connective tissue that is highly vascularized and aids in the overall structural support for the body [5]. Along with structural support, the vascularized nature of bone allows it to mobilize vital nutrients and minerals that help to support osteoprogenitor cells that ultimately form new bone tissue during healing after an injury. Bone tissue is an extremely unique tissue in the sense that it is comprised of two different components, cortical and cancellous bone [6]. Cortical (also known as compact) bone is the hard, dense outer surface of the bone that surrounds the inner cancellous bone (sometimes referred to as trabecular bone), which is the spongy, vascularized inner portion of bone tissue. 

Currently, natural bone tissue grafts are viewed as the gold standard for treating bone injuries because of the key factors that preexist within these grafts. Natural bone grafts innately exhibit both the physical and biological characteristics needed to promote new bone formation after a traumatic injury. These replacements include both autologous grafts in which tissue is taken directly from the injured patient and allogeneic grafts, where donor tissue from another patient is used. However, like with many tissue transplant procedures, there are inherent disadvantages associated with these techniques; with autografts, donor site morbidity remains a large concern, while with allografts, graft rejection or inflammatory responses due to the foreign tissue implants are a possible outcome [7]. These limitations, along with the increasing number of non-unions being reported, have further increased the need to study and define new alternative sources of materials to be used as bone tissue substitutes. 

Due to this, tissue engineering, more specifically bone tissue engineering (BTE) has become one of the most widely investigated and most intriguing fields of biomedical engineering. Within this field, scaffold-based BTE has significant potential to alleviate many of the limitations associated with bone grafting, bringing a vast amount of promise to the future of bone defect therapies. Traumatic bone injuries still yield challenges clinically in orthopedics, including the need for invasive surgeries to reconstruct proper support and functionality to regions of largely defected bone [8]. The invasiveness of currently used surgical grafting techniques often leave unsatisfactory results which come with substantial social and economic encumbrances for the patients that suffer from these types of disorders [9,10]. 

The primary goal of tissue engineering is to create operative replacements for damaged or diseased tissues [11]. For BTE specifically, scaffolds are used as temporary replacements that not only support the regeneration of lost tissue, but also in most cases help keep the native bone structurally sound. The ideal scaffold for any bone tissue application would consist of a biocompatible, biodegradable, and a reasonably supportive material that would lack any form of cytotoxicity [12]. There are, however, many other factors that play a role in deciding which materials would be best suited for bone scaffolding applications, for example, from a surgical implantation standpoint, a scaffold would ideally be flexible and easily manipulated [13]. These factors, in conjunction with several other necessities play a fundamental role when deciding on the ideal material or materials to replace existing bone tissue grafts.

The loss of bone whether over time or abruptly will negatively affect the quality of a person’s life [14]. With the increased frequency of bone grafting procedures several advancements have been made within this field. Many of these advances stem from the numerous discoveries in the use of “smart” biomaterials as substitutes for bone tissue. Natural bone grafts exhibit all the required factors needed for new bone development; osteoprogenitor cells, a three-dimensional (3D) porous matrix for cellular support, bone morphogenic proteins (BMPs), and other growth factors to promote bone regeneration [15]. With the idea of using “smart” biomaterials as hard tissue replacements, synthetic scaffolds can now possess these same key factors while providing better surgical and cosmetic outcomes which could substantially improve high-risk orthopedic reconstructive surgeries [16].

For decades, the BTE research landscape would suggest that scaffolds with biological and structural properties, most closely resembling healthy bone, would have the greatest chance to replace natural grafts; however, recent studies involving external stimulation and conductive scaffolding materials have shown otherwise [17,18,19]. Bone remodeling is not only manipulated by the biological additives and the physical characteristics of scaffolds, but also by other aspects such as physical and chemical cues or signals that are generated by external factors. Conductive bioactive materials are increasingly becoming more plausible as future synthetic scaffolding substrates due to physiochemical properties that could potentially enhance bone regeneration through certain external factors, like cyclic mechanical loading. This phenomenon can be described through Wolff’s Law, or the ability to transform mechanical loads into electrical currents [20,21]. With this type of adaptive scaffolding technique, normal daily activities could generate the signals required to not only enhance cellular attraction to or near the implantation site, but also the development of new, healthy bone tissue over time. Researchers have further demonstrated the idea of using conductive materials and stimulation to enhance cell differentiation and bone formation in several studies [18,19,22,23].

The purpose of this review is to highlight the growing need to produce biologically-relevant bone tissue substitutes, as well as the necessary biological and structural requirements for these substitutes. The current state of BTE including the cells, materials, and cellular cues used will also be highlighted. Lastly, the potential for conductive materials to be used as synthetic bone grafting replacements will be discussed.

## 2. Biological and Structural Requirements for Engineered Replacements

### 2.1. Biological Requirements

Biocompatibility is considered to be the most essential quality that any materials and the byproducts associated with biomaterials must have when being considered for use as potential tissue replacements. Bone scaffolding materials should not only induce osteogenesis, but also prevent adverse effects, such as the reduction of the healthy, native tissue when implanted in vivo. All implanted materials will initially be seen as foreign by the body and generate an inflammatory response; therefore, ideal BTE scaffolds should be non-cytotoxic, minimizing their generated immune response when implanted [24,25]. With this in mind, bone scaffolds should be held to strict sterility constraints, while consideration is made for the effects different sterilization methods may have on other aspects of scaffolds, such as structural integrity [26]. The ability for cells to adhere to scaffolding surfaces is another important aspect when determining the use of different materials as bone replacements. Furthermore, any material used as a bone substitute should possess osteoconductive properties, allowing for bone forming cells and additional osteoinductive elements (i.e., vasculature) to migrate across and into scaffolding to generate new tissue [25,27]. In addition, with scaffolds serving as temporary placeholders or replacements that support new tissue formation as they degrade, scaffolds should be bioresorbable as well as controllable in their degradation rates.

Some of the most challenging aspects that exist with the creation of these scaffolds are associated with balancing their strength and integrity with the necessary biological markers and cues that are needed for cell recruitment and bone tissue ingrowth overtime. Scaffolds can be created with ideal mechanical properties that are comparable to natural bone (i.e., those constructed of magnesium), but can, alone, lack essential requirements like an easily controllable degradation rate in order to complement tissue development and maintain structural support [28]. Furthermore, the use of slowly degrading metals, such as steel or metal alloys like Ti and Co-Cr as implants can be linked to stress shielding and ultimately to the loss of bone mass [29]. As previously mentioned, in most cases, bony scaffolding should be made to be biodegradable and bioresorbable so that the body can naturally breakdown the contents of the material as well as begin to absorb them within the body. This is because when scaffolds are implanted in vivo and begin to degrade as new bone forms, there could be a discharge of harmful byproducts that could lead to changes in pH levels at or near the implantation site. Changes in pH could result in decreased cell recruitment, slowed cell proliferation and ultimately a lack of bone formation over the long term. To combat this, many researchers deem it necessary to create scaffolds based on the composition of normal, healthy, living bone tissue.

Bone is composed of collagen, water and several minerals that come together to form the bone matrix which is then separated into two parts, the organic and inorganic matrix. Within this split matrix there consists of about 70% hydroxyapatite (HA) found in the inorganic portion and 30% collagen found in the organic portion by weight of the total bone [30]. Scaffolds should possess similar biological profiles as natural bone while limiting potentially toxic shortcomings. Oftentimes it is difficult for single materials to mimic the composition of natural bone tissue, thus, creating the need to implement several different materials, coatings, and composites in order to closely replicate bone tissue biologically.

### 2.2. Structural Requirements

Bones provide structural support for the human body and act as a reservoir for many essential minerals [31]. This specialized tissue possesses a complex hierarchical structure different than any other tissue in the human body spanning several different length and width scales [32]. These different scales include macro (cortical, or compact bone and cancellous, or trabecular bone), micro (consisting of haversian canals, osteons, and concentric lamellae), and nano (collagen fibrils, HA crystals and other minerals). 

Bone is an anisotropic tissue which presents challenges structurally because of this fact alone. Bone tissue has been measured to show both higher compressive strengths as well as elastic moduli when measured longitudinally compared to measurement in the transverse direction, making bony scaffolds even more challenging to create [33]. When constructing an implantable scaffold for bone tissue one must look at the overall supportability as well as the ability of scaffolding to function as normal human bone when subjected to everyday compressive loading. These factors require scaffolds to be strong in a sense of support, but also flexible enough to prevent shear fracture when compressive forces are applied with daily functions.

While strength is a very important factor that needs to be carefully considered when thinking of creating a bone-like scaffold, other scaffolding components such as porosity play a more essential role in the regenerative success of scaffolds as well as an additional determinant to overall scaffold strength [34,35]. This further complicates the idea that a scaffolds ability to act alone in structural support should be deemed a necessary function, particularly when compared to scaffolds designed with microstructures closer to natural bone tissue. Porosity is defined as the percentage of void spaces within a solid, so as porosity is increased structural integrity will simultaneously become more compromised [35]. Porosity is necessary for the formation of new tissue because it allows for cell migration as well as angiogenesis leading to the delivery of key nutrients [34]. Similar to porosity, surface morphology can negatively affect the overall strength of a bone scaffold but will be beneficial to the ingrowth of new tissue. Surface modification and additive coatings can aid in the attachment of new cells and the integration of the implant into the injury site. 

Scaffold strength is predominantly influenced by the internal structure of the graft and must be designed with such in mind. Bone scaffolding materials as well as their overall architecture, including surface characteristics can play a huge role in the strength of scaffolds, but with recent research there has been more of a decline in the idea of strength being the most highly valued scaffolding requirement and an increase in osteoconductive capacity being the leading necessity of boney scaffolding [36]. While strength is key for load bearing bone grafts, the ability for cells to attach to, spread across, and deposit new mineral in place of bone scaffolds is among the most desirable outcomes for BTE constructs.

## 3. Bone Tissue Engineering: Cells, Materials and Cues

**Figure 1:**
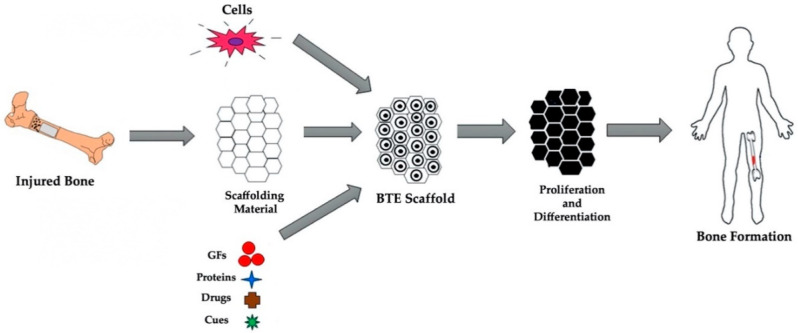
Typical bone tissue engineering approach. Bone defects are commonly healed through the use of scaffolds which are comprised of osteoprogenitor cells, relevant biomaterials and biochemical cues, such as growth factors. Figure modified from reference [38], with permission from Elsevier (License Number 5198230358722), 2021.

In BTE, biomaterials are used as temporary substrates that provide an ideal microenvironment for cells to attach, proliferate, differentiate, and potentially generate new bone tissue where an injury has occurred [37]. Within bone tissue scaffolds a 3D porous matrix consisting of cells, biocompatible materials and essential biological and biochemical cues are present. The typical process for scaffold-based approaches for BTE can be seen in Figure 1.

### 3.1. Cells

Bone regeneration is a process that begins with the recruitment of cells to a site due to inflammation and other natural body responses caused by an injury. Cells will then expand, form their own extracellular matrix (ECM), differentiate, and ultimately deposit the makings of new, healthy bone tissue where a void is present. Most BTE approaches typically deal with mesenchymal stromal cells (MSCs). These multipotent cells can be found in many different soft and hard tissue sources throughout the body, including bone marrow and stored fat. Bone marrow-derived stromal cells (BMSCs) and adipose-derived stromal cells (ADSCs) are typically used in BTE but provide different advantages and disadvantages.

BMSCs are the most frequently used cells in BTE applications due to the many favorable regenerative characteristics this stromal cell population displays [39,40]. MSCs isolated from bone marrow were first characterized as a plastic-adherent cell population exhibiting a fibroblast-like appearance in monolayer culture during the late 1960’s [41,42]. BMSCs have been described to have high osteogenic potential, both anti-inflammatory and immune-modulatory properties, as well as the ability to stimulate angiogenesis [40,43,44]. The widespread use of these cells for BTE applications also leads to familiarity with anticipated in vivo responses, further promoting the use of BMSCs for use in scaffolding approaches. BMSCs have also been shown to release paracrine factors into their surrounding microenvironment that could lead to increased fracture healing through the recruitment of macrophages [45]. However, difficulties in harvesting BMSCs from marrow and poor long term multipotency with increased passage have proved to be a challenge in clinical use due to necessary cellular expansion in vitro.

ADSCs have been targeted as useful cells for BTE due to their ability to differentiate into several different types of cell lineages, their ease of accessibility, their immunogenic capabilities and the stability they have shown in long term cell cultures [46]. ADSCs have generally been known to be harvested from fat using minimally invasive techniques. These stromal cells also possess several key factors that make them ideal for BTE, including their ability of self-renewal, inherent plasticity, and their abundance [46,47]. ADSCs have high angiogenic properties as well, indicating higher potentials for vascular ingrowth and nutrient deposition to bone forming cells [48,49]. ADSCs also provide an incredible source of biological factors that are released via exosome secretion which can aid in the signaling of more bone forming cells [50]. These cells are broadly multipotent and have far less ethical concerns when compared to embryonic stem cells (ESCs) as well [51].

### 3.2. Materials

The complex properties of bone have created the need for engineered substitutes for bony implants to take on several different approaches. Of such, scaffolds comprised of either polymers, ceramics, metals and in most cases, some combination have been primarily used as base materials for scaffolding. Though each material brings necessary properties that are vital to bone tissue, there is still a great deal of work to be done in order to find an ideal scaffolding material that will both be biologically relevant and have structural similarities to healthy bone tissue. This ideal material must allow for scaffolds to be biocompatible, biodegradable, highly porous, easily manipulated, osteoconductive and ultimately able to lead to osteogenesis while mechanically suitable to bear relevant loads [24,27,52]. While bone tissue replacements have normally been thought to require strengths that mimicked natural bone, recent findings suggest other biological cues play a more vital role.

Usually, one material will not exhibit all of the necessary properties that are prevalent to bone, thus creating the need to combine and manipulate these materials in order to gain the desired characteristics. The properties of certain types of materials are more applicable to being biocompatible to bone more so than to being structurally supportive enough to be used as stand-alone implantable scaffolds. Materials such as collagen might not be used as base materials but are oftentimes used as coatings or additives to improve the mechanical properties of scaffolds as well as the osteogenic response of cells that are seeded onto scaffolds [53,54]. Additionally, coatings are used to combat toxic qualities of certain compounds found in scaffolds as well. Collagen and HA are some of the most common coatings that are used in bone tissue scaffold creation because of how predominant they are within natural, healthy bone tissue. 

Currently, scaffolds are being fabricated in order to not only be deemed biocompatible, but also act as vehicles to transport cells and growth factors [55]. These additions to current scaffolds will ultimately allow them to have improved properties while maintaining biological necessities. For example, while metal-based scaffolds show greater overall structural stability when compared to polymer-based scaffolds, they tend to lack the required biological traits of natural bone. By incorporating biologically relevant coatings onto metal-based scaffolds, improved biocompatibility and osteogenic differentiation of cells can be achieved while maintaining their given physical properties [56,57].

#### 3.2.1. Polymers

Polymeric materials have been studied and used as functional scaffolding materials because they exhibit the most controllability when used as 3D printed scaffolds [58]. These polymer-based materials are seen as the most practical base materials for bony scaffolding simply because of their printability, or ability to be customized into desired geometries. Polymers are not hard to control and can form scaffolds of various porosities relatively easily. 

There have been many types of polymeric materials used for BTE. These polymers can either be classified as naturally derived, such as collagen and gelatin or synthetic polymers, such as polylactic acid (PLA), polyglycolic acid (PGA), and their copolymer poly(lactic-co-glycolic acid) (PLGA) [59]. Natural polymers can also be separated into three distinct groups: proteins (i.e., collagen, silk, and fibrin), polynucleotides (i.e., DNA and RNA), and polysaccharides (i.e., chitosan, cellulose and glycosaminoglycans or GAGs) [60]. Polymers are further defined as either degradable or nondegradable depending on their intended uses, whether that be for long term implantation or dissolvable grafts [61]. For instance, degradation of PLA, PGA and PLGA are ranked with the following order; PLGA > PGA > PLA in order from longest to shortest degradation time [62]. 

Naturally derived polymers are frequently used in BTE due to their biological recognition when used as implants. Natural polymers also tend to boast high cellular adhesion rates and provide excellent support for ECM deposition [63]. While this is a great advantage, when using naturally derived polymers in bone scaffolding there are drawbacks such as immunogenicity, or induced immune responses due to impurities that could be present within the polymer [64]. Synthetic polymers, on the other hand, have been commonly used in BTE due to a wide range of chemical and physical properties as well as the ability to be easily shaped into complex geometries. However, many synthetic polymers that are used in the creation of bone scaffolds, specifically PLA and polycaprolactone (PCL), which are among the most commonly used, tend to be hydrophobic, leading to the need for surface treatments, coatings, or the incorporation of other materials to aid in cell attachment [65,66].

Overall, polymers are seen as the most adaptive material to use when making scaffolds. These materials can be manipulated in ways that others cannot and have shown suitable qualities relative to biocompatibility and cell adhesion. While polymeric materials have been investigated for BTE and are widely available, no single polymer can meet all the vital requirements that are needed in bone replacement scaffolds. These polymers can further be improved with the addition of other materials in order to help maximize some of the scaffolding requirements that they lack, in some cases. Commonly used synthetic and natural polymers for BTE applications along with relevant advantages can be seen in Table 1.

#### 3.2.2. Ceramics

Ceramics have been investigated as materials for bone scaffolds for many reasons, but none more significant than their biocompatibility. Bioceramics nearly mimic bone tissues mineral composition; they provide the highest cell attachment and growth for osteoprogenitor cells when compared to other materials [77,78]. Generally, due to these factors, ceramics react very well when tested in vivo because of the similarity to healthy bone tissue. These materials tend to bond directly with the surrounding living tissue near implantation sites which is an extremely important factor when considering ceramic biomaterials for scaffolding purposes [79]. Commonly, bioceramics can be categorized as three main types: bioinert (i.e., alumina and zirconia), bioactive (i.e., HA and Bioglass^®^), and biodegradable (i.e., tricalcium phosphate and calcium sulphate). Some of the most utilized bioactive ceramics in BTE include calcium phosphates (CaPs) such as HA, calcium sulfate, calcium carbonate, and tricalcium phosphate (TCP) in addition to known bioactive glasses (BAGs) like 45S5 Bioglass^®^ [80,81]. 

While bioceramics fundamentally are more biologically in tune with bone when compared to polymers; these materials lack the tensile and torsion strengths that are comparable to natural bone thus limiting their ability to be used alone as BTE scaffolding base materials [82]. Furthermore, many ceramics are less bioresorbable than the polymers used in BTE, which could be detrimental to new bone ingrowth [83]. Due to this, researchers have resorted to the manipulation of slowly degrading ceramics, particularly HA, by structure destabilization (through the addition of ions) in order to accelerate degradation [84]. Table 2 provides a short list of ceramics that have been used for BTE applications along with their inherent strengths and weaknesses.

#### 3.2.3. Metals

Metallic materials exhibit the best physical properties when studied as bony scaffolds. These materials have been used as implantable devices for many years and are known for their mechanical strengths when used in this manner. Metals and their alloys, such as stainless steel, titanium (Ti), and cobalt (Co) are some of the most reputable metals used in scaffolding approaches because of their biocompatible traits, superior corrosion resistance, and mechanical strengths, a property that had often been seen as the top requirement for bone scaffolding materials until recently [92,93]. 

Depending on material properties, some metals can be too weak to be worked into geometries with desired porosities, while others may result in scaffolds that are too brittle to function alone when shaped into some of the complex architectures required for bone scaffolding [94]. Frequently, metals are used in the form of plates as supports when bone defects require stabilization in order to heal properly; this technique, plate osteosynthesis or internal plate fixation dates back over 100 years [95]. Although this technique does return the injured limb back to functionality quicker than without the support, limitations to this technique include the use of painful hardware (i.e., screws, nails, and wires) and the potential for misalignment of fractures as they heal due to the rigidity of the given supports [96].

While metals do show mechanical properties that are most comparable to bone tissue when comparing them to other scaffolding materials, they are frequently seen as being too stiff relative to natural bone, resulting in stress shielding and ultimately failure of implants [97]. Furthermore, while certain metals are considered to have decent biocompatibility and the availability of metals and alloys are remarkable, very few metals actually fit the requirements to be used as functioning biomaterials on their own [98]. Several factors play a role in this reasoning, for one, most metals release toxic byproducts when they degrade and can cause damage to preexisting bone tissue [99]. Furthermore, the degradation rates of most metals have been found to be difficult to control or estimate resulting in metals being an unideal bone scaffolding base material.

#### 3.2.4. Composites

Due to the need for engineered bone scaffolding to possess numerous biological and physical characteristics (i.e., biocompatibility, biodegradability, bioactivity, a porous architecture, and have suitable mechanical properties) that simply cannot be met by using a lone polymer, ceramic or metal, composite materials have been tested [12]. Composites combine two or more materials with varying physical and biological properties together to create new, specialized materials that show improvements to several key components related to the integration of scaffolds into native bone tissue (i.e., improved cellular attachment and scaffold bioactivity). 

The process of combining biomaterials together has been used in order to enhance the mechanical and biological properties of different scaffolding materials as well as to improve the processability of certain scaffolds. Composites for bone tissue have taken on several different matrix compositions, whether that be polymer-based, ceramic-based or metal-based composites [100,101,102]. Functional composites have also been created using conductive materials in order to respond to external stimulation; these functional composites form scaffolds that have been shown to have greater protein absorption as well as enhanced cellular adhesion and proliferation when compared to non-stimulated scaffolds [103,104]. 

Within BTE, studies have demonstrated improvements to the mechanical strength and cellular attachment of polymer-based scaffolds, the bioactivity and osteointegration of metal-based scaffolds, and the toughness of ceramic-based scaffolds by combining various biomaterials [56,100,105,106,107]. The use of composite materials has greatly improved several physical and biological aspects of engineered tissue replacements; however, the complexities of natural bone tissue have still not fully been recreated in a laboratory setting. 

### 3.3. Biophysical and Biochemical Cues

Evaluation of the development of new bone tissue shows that this process can be largely attributed to the presence of biophysical and biochemical cues within the surrounding microenvironment. Biophysical cues such as porosity and substrate stiffness have enabled BTE scaffolds to better regenerate tissue following injury and other losses of natural bone, as well as to determine MSCs lineage fate in vitro [108,109]. Upon implantation, scaffolds come into direct contact with native tissue; their structure and surface characteristics play a tremendous role in their overall integration within the body [110,111]. Through the addition of biochemical cues (i.e., growth factors and bioactive molecules like nitric oxide) into BTE scaffolding, faster regeneration of healthy bone tissue is possible [112,113]. However, poor administration of growth factors (i.e., uncontrolled release from scaffolds) can lead to ectopic bone growth [114]. Alternatively, recent studies have shown that biophysical cues, particularly scaffold conductivity, in conjunction with electrical stimulation (ES) can better aid in the generation of healthy bone tissue without the need for additional growth factors [18,115]. Furthermore, by employing stimuli-responsive (i.e., piezoelectric) materials as scaffolds for BTE applications, cells can convert scaffold generated stimuli into biochemical signals that illicit changes to other cellular signaling events, potentially providing scaffolds that closely approximate natural bone tissue.

## 4. Piezoelectric Effect in Bone

First discovered in 1880 by Pierre and Jacques Curie, piezoelectricity is the ability of certain materials to generate electricity due to deformation. Naturally, bone exhibits piezoelectric potential, converting applied mechanical stresses into electrical currents within itself. This innate ability has been studied experimentally, both in vitro and in vivo using various piezoelectric biomaterials and modes of deformation as a means to prompt cells to migrate, as well as to promote greater deposition of bone forming minerals [116,117,118]. This phenomenon has also been used as an effective adjuvant in many ES therapies to enhance bone regeneration [119]. Many within the field of tissue engineering generalize this idea using Wolff’s Law, an oversimplification in the case of this extremely complex tissue, which simply notes the bone’s ability to adapt due to mechanical stresses. While widely known and accepted as true by both scientists and clinicians, piezoelectricity in the form of a scaffold guided approach is not traditionally considered as an alternative, however, this methodology could prove advantageous in the development of a new generation of biomimetic substitutes for bone tissue.

As early as the 1950’s (and potentially even earlier), researchers reported the generation of electrical potentials in bone tissue from mechanical deformation, leading to the first speculations of the relationship between these strain generated potentials (SGPs) and mechanically induced bone remodeling (i.e., increased bone density with exercise) [20,21,120,121]. Soon after, studies emerged detailing the difference in piezoelectric potentials for wet and dry bone, in addition to research describing the more physiologically relevant response bone has to loading based on Biot’s theory of dynamic poroelasticity [122,123,124,125,126]. These findings improved upon previous notions surrounding the piezoelectric effect in bone by moving away from the oversimplified models from the past regarding mechano-electric SGPs and towards a more logical understanding of this phenomenon.

The first experimental evidence detailing specific cellular mechanisms involved in mechanically induced bone remodeling was reported in the early 2000’s; the role that voltage-dependent calcium (Ca^2+^) plays in bone remodeling was determined, linking SGPs to specific cellular responses leading to bone formation [127]. Voltage-dependent Ca^2+^ channels are key in the conversion of changes in membrane potential (i.e., mechanical deformation or depolarization) into intracellular Ca^2+^ signaling, bridging the gap between external forces on bone tissue and the resulting biochemical signals generated by cells [128,129,130]. Intracellular Ca^2+^ play a structural role in bone tissue and a rapid rise in Ca^2+^ is one of the earliest detected responses of a mechanically activated bone cell [131,132]. During bone repair, Ca^2+^ ions are released into the extracellular environment resulting in Ca^2+^ being constantly available for several biological functions of osteoblasts and osteoprogenitor cells, thus making intracellular Ca^2+^ signaling a key to bone tissue repair and regeneration. Intracellular Ca^2+^ mobilization is triggered by several different forms of mechanical stimuli, such as membrane straining, increases in pressure and fluid flow-indued shear stress, which will cause intracellular Ca^2+^ in osteoblastic cells to increase as shown with in vitro studies [133,134,135,136]. The progress made in the understanding of specific mechanisms involved in bone cell mechanotransduction gives a solid foundation for the use of conductive and piezoelectric scaffolding materials for BTE applications.

**Figure 2:**
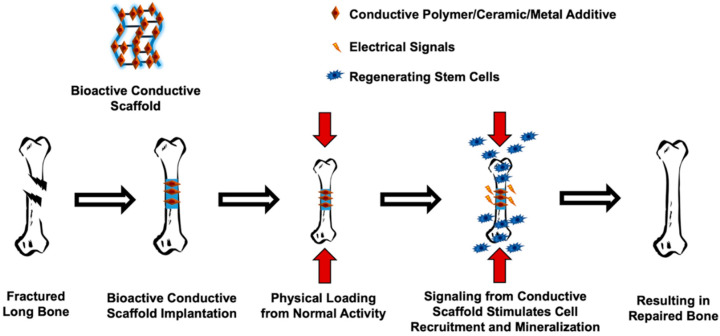
Proposed piezoelectric effect in bone in conjunction with bioactive conductive scaffolding. The implanted bone scaffold under compressive stress generates negative and positive electrical dipoles. Osteoblasts are then attracted to the negative dipole where they generate new ECM and deposit minerals that form new healthy bone tissue.

By weight, naturally dense bone is comprised of nearly 30% collagen type 1 and 70% HA as stated previously [30]. Piezoelectric potential is prompted by these two organic and inorganic materials. When applied stresses create local potential gradients along collagen fibers the surrounding particles become charged [21,137]. These particles then travel towards the surface of the bone or synthetic grafting material in this case and offer stimuli for osteoblasts that form new bone. Figure 2 below depicts the proposed mode of action for how piezoelectric, conductive scaffolding can exploit the mechano-responsive properties of natural bone tissue in vivo.

While the piezoelectric potential of conductive materials has not widely been perceived as a method that could be used in order to enhance scaffold-based bone tissue implantable grafts it is a new and more increasingly studied approach. With this type of methodology, scaffolds can become less like temporary stents and more like living, normal bone tissue with adaptive functional implants.

## 5. Conductive Materials and Strategies for Induced Bone Regeneration

### 5.1. Application of Conductive Materials

Conductive materials are increasingly becoming some of the most studied “smart” biomaterials in the field of tissue engineering. These types of materials are generally found as additives within 3D tissue scaffolding. These conductive scaffolds can then be divided into two distinct categories based on their composition, conductive polymer-based scaffolds and conductive nanomaterial-based scaffolds. Conductive bone scaffolds have the ability to transfer both electrical and electromechanical signals directly to targeted cells; showing the ability to improve proliferation and osteogenic differentiation in vitro, as well as bone formation in vivo, as presented in several studies [22,77,104,115,138]. Through this, conductive scaffolds can be seen as a way to not only decrease healing times associated with traumatic injuries, but also improve tissue repair overall. Furthermore, conductive scaffolds could alleviate one of the main drawbacks associated with current tissue grafting by presenting a more readily available option to patients.

Conductive polymers (CPs) were first introduced over four decades ago when a several millionfold increase in the conductivity of polyacetylene (PAc) was observed after oxidizing the polymer using iodine vapor [139,140]. During this redox process, referred to as doping, charge carriers are formed within the polymers, subsequently giving them their high conductivity [141]. These organic materials possess both magnetic and electrical properties, combining the flexibility and ease of processing found in polymers with the electroconductivity of metals and semiconductors [142]. Moreover, CPs are more chemically diverse than their inorganic counterparts, having the ability to be more resistant to corrosion as well as increased tunability, with respect to conductivity [141]. 

The production of conductive polymers is mainly done through two methods, either chemical or electrochemical synthesis [143]. Generally, the main difference between these two synthesis techniques is the resulting form of the CP; electrochemical synthesis produces CPs as thin films while chemical synthesis can produce thick films or CPs in power form, making this method more promising commercially [143]. While chemical synthesis enables the ability to create novel CPs, it has been shown that using chemical synthesis methods produce a polymer with less conductivity when compared to electro-synthesized polymers [144]. To date more than 25 CP systems exist with polypyrrole (PPy), polyaniline (PANI) and poly(3,4-ethylenedioxythiophene) (PEDOT) being some of the most studied [145]. 

PPy is a conductive conjugate polymer with great mechanical, electrical and stimuli-responsive properties, making it one of the most suitable for use in biomedicine [146]. It can also be synthesized with various different bioactive molecules to increase its feasibility for use in scaffolding as well as proven high electrical conductivity under biological conditions [147,148]. PANI may be the second most studied CP and offers several advantages over other CPs, including its low-cost, ability to be easily synthesized and being able to electrically switch between conductive and resistive states [149,150]. PEDOT, a derivative of polythiophene (PTh), possesses both high chemical and electrical stability in addition to having better thermal stability than PPy [151,152]. Other current CPs used in research are listed in Table 3.

As stated previously, biocompatibility is considered to be the most essential quality that biomaterials must have when being considered for use as potential tissue replacements. It is worth noting that several conductive polymers including PPy, PANI, PTh, and PEDOT have been shown to support cell attachment and proliferation with various cell types for different tissue engineering applications, which is an essential aspect of the biocompatibility of a material [153]. Bone tissue is naturally conductive (for list of conductivity values see Table 4). The use of conductive materials for BTE applications can allow for a more electrically similar, biomimetic scaffold. Through the incorporation of CPs such as PANI and PPy, researchers have been able to produce scaffolds with increased conductivity, these values have been similar to cortical and cancellous bone tissue in some cases [154,155]. Additionally, the performance of conductive polymer-based scaffolds, in terms of bone formation, can further be enhanced through exogenous ES (i.e., direct current and capacitive coupling), specific examples presented in the following section [156].

Conductive nanomaterials can be found in many different forms, including nanotubes, nanosheets, nanoparticles, and nanowires [158,159,160]. Some of the main conductive nanoparticles and nanowires are composed of gold, silver, copper, and aluminum [161]. Carbon nanotubes (CNTs) and graphene nanosheets have also been studied extensively for bone regeneration [158,162,163]. To date, conductive nanomaterials have been used to create biosensors, to deliver drugs and in many ways throughout tissue engineering to improve engineered tissue replacements [164,165,166].

It is well documented that characteristics such as surface roughness and wettability (whether or not a material is hydrophobic versus hydrophilic) play a huge role in cellular attachment to given scaffolds [167,168,169]. However, in the creation of composite scaffolds, it is not always possible to achieve ideal characteristics in relation to surface properties. By using conductive nanomaterials as additives, thin films and functional coatings for scaffolding, improved characteristics can be attained. Researchers have shown that conductive carbon-based nanomaterials, such as graphene oxide (GO), have been able to improve hydrophilic surface properties of commonly used synthetic polymers when incorporated into scaffolds, increasing the attachment potential of cells [170]. The reduction in contact angle can be attributed to an increase in hydroxyl functional groups as the percentage of GO rises within the scaffold. 

By incorporating conductive nanomaterials into scaffolding matrices, particularly CNTs, a variety of structural scaffolding characteristics (i.e., strength and flexibility) can be improved as well as the induction of angiogenesis, the reduction of thrombosis and the manipulation of gene expression for tissue repair [171]. Furthermore, studies have shown that by increasing the percentage of CNTs in scaffolding, bone scaffolds can achieve a significantly higher compressive modulus, while also improving bone formation in vivo with exogenous ES [115,172].

### 5.2. Strategies for Induced Bone Regeneration: Electrical and Mechanical Stimulation

The incorporation of conductive materials into the development of scaffolding for bone is of significant interest due to the electrical properties this tissue [153,156]. The use of conductive materials can not only improve several aspects of tissue regeneration but specifically improve cell-scaffold interactions such as adhesion and proliferation [138,173]. An increase in scaffold surface conductivity could lead to more efficient absorption and deposition of serum proteins, which would aid in cell attachment and proliferation [174]. It is also documented that ES can effect cell orientation as well as cell adherence to surfaces [175]. With conductive biomaterials, ES to cells can be enhanced, further improving cell-scaffold interactions.

In addition to overcoming cellular attachment limitations, current approaches have needed new ways to further improve bone tissue ingrowth. Electrical and mechanical stimulation have been used within experimental settings in vitro, as well as with therapeutic rehabilitation in vivo for improved outcomes involving bone regeneration with positive results [176,177,178,179]. It has been shown that electrical conductivity within the 3D matrix of scaffolds promotes better tissue response when compared to induced stimulation through different mediums [180]. Additionally, ES has been shown to increase osteogenic differentiation and have an influence on the behavior of electroactive tissues, like bone as presented previously [19,23,181]. Within BTE, conductive scaffolds centered around polymers, hydrogels, composites, and nanofibers have been utilized [22,138,153]. 

Many of the materials reported previously have been widely applied to tissue engineering of muscle, cardiac, and other tissues, but specifically with regard to bone, graphene nanoparticles and polymers, such as PANI and PEDOT have been studied significantly. Of the work reported in current literature that has used conductive scaffolds with different compositions, specifically for bone, improvements such as scaffold characteristics including elasticity, surface roughness, and electrical conductivity have been reported as well as improved cellular adhesion, proliferation, and differentiation [138,154,182,183]. The addition of ES does however further improve upon the cellular response of given conductive scaffolds alone. Currently, conductive fibers, particularly electrospun fibers, have been the most abundantly studied. This is partly due to the ease of scaffold creation and the ECM like mat structure that is formed through this process [184]. Studies have shown that nanofiber scaffolds containing conductive nanoparticles made of PANI and graphene were able to increase cell viability with increased amounts (up to 2% in PCL) [185]. Suggesting that different cellular functions could be improved through conductive nanomaterial incorporation, although there are limitations. However, 3D printed polymers do present the greatest advantage in BTE due to the tailorable geometries that they present, allowing for tissue replacements that fit directly into voids caused by traumatic bone injuries.

Electrochemical reactions in natural bone tissue create dipoles that form in response to normal loading on the tissue. This causes electroactive bone tissue to adapt structurally. Electrical stimulus has been used to treat bony injuries clinically for nearly 50 years and has been studied extensively in vitro [186,187]. The main cells that uptake these signals are osteocytes, which are mature bone cells entrapped within the matrix left behind after the creation of new bone tissue. Osteocytes are the most abundant cell found within bone, outnumbering both osteoblasts and bone absorbing osteoclasts by nearly 20-fold. These cells then act as antennas, picking up electrochemical cues and transporting them through their functional network as intracellular Ca^2+^ signals [188]. Researchers have replicated these signals in vitro showing the ability of conductive materials to be used for future synthetic scaffolding [189,190].

ES can be administered to cells in several ways, which include capacitive, inductive, and direct current ES. Capacitive ES delivers an electric field through the target, inductive ES delivers stimulation via an electromagnetic field generated by the current flowing along the solenoid, and direct current ES delivers an electric field and current flow through the target [191]. These ES techniques have been shown to enhance osteogenesis by promoting pro-osteogenic protein (i.e., BMP-2, osteopontin, COL1 and ALP) expression, however, despite these claims, the mechanisms of electrically induced osteogenesis are not fully understood [191,192,193]. In conjunction with conductive materials, the ES to cells could further be enhanced due to less restrictive current flow (in the case of direct ES) and may prove beneficial. Studies have shown that capacitive ES triggers Ca^2+^ influx in bone cells, and inductive ES induces Ca^2+^ release from intracellular storage, resulting in osteoblast proliferation [127]. The combination of electrically conductive scaffolds (PPy incorporated into PCL) and direct current ES has also been shown to significantly enhance mineral deposition of ADSCs compared to conductive scaffolding alone [194]. When ADSCs were exposed to ES with blockers of voltage-dependent Ca^2+^ those improvements were nullified, indicating that ion fluxes through these channels, which are activated by ES, induce different cascades of reactions in ADSCs, concluding that voltage-dependent Ca^2+^ channels play a more critical role than sodium, potassium, and chloride.

The piezoelectric potential in bone tissue further drives the need to study more conductive materials as potential replacements for natural and currently used synthetic bone substitutes. It has been noted that without usage, bone tissue will only develop to between 30 and 50% of its normal mass [195]. This is because osteoblasts are mechanosensitive, meaning that they are able to sense and respond to biophysical factors within the microenvironment [196]. When these biophysical cues are not sent out to cells, a reduction of bone mass will occur. This phenomenon has prompted researchers to take advantage of this cellular trait and study the effects of mechanical loading on cell differentiation in vitro [197]. Research suggests the ability of conductive scaffolds to not only recreate bone’s natural ability of self-renewal but even speed healing times in certain cases through the enhanced stimulus discussed [198,199].

### 5.3. Advantages and Disadvantages

Conductive materials have been able to produce greater cellular response as well as improved osteogenic differentiation of certain cell lines in vitro. Nonetheless, as with any new ideas that contest existing approaches, bioactive conductive materials as BTE implantable grafts come with certain challenges. Research has shown that conductive materials can be used as functional adaptive tissue replacements, but there is still work that needs to be done to prove long term conductive scaffolds as the most viable option for new bone development.

The use of conductive scaffolds could allow for a more natural tissue like response to certain mechanical and electrical stimulus as well as a higher integration potential with healthy native tissue. However, it is extremely important to optimize the composition of conductive scaffolding based on the cytotoxicity threshold (maximum amount of conductive material that will remain nontoxic to cells). Studies have indicated that while some CPs like PEDOT and PPS prove beneficial to cell viability with increased concentrations, excessive amounts of PANI can have a cytotoxic effect on cells in vitro [22,154]. Furthermore, studies reported in the review on the applications of CNTs in BTE by Pei et al. [158], suggest that the cytotoxicity of CNTs is influenced by many factors relating to their size (i.e., length, surface area, and diameter). The use of stimulus in conjunction with conductive materials does show favorable results when compared to conductive materials alone, however, electrical stimulus can be detrimental as well if not properly administered in vivo [200,201]. By simply applying direct current voltages maintained in vitro, in vivo electrical stimulation can induce larger currents in live tissues due a much lower 3D bulk resistance compared to electrotaxis chambers used [202]. Theses higher currents produce more heat, which cannot dissipate effectively as well as significantly more electrode bioproducts (i.e., changes in pH) that are toxic to cells [201].

Overall, conductive materials as bases for scaffolding could take advantage of the piezoelectric potential found in healthy bone tissue while creating a substrate for cells to proliferate at faster rates than currently used synthetic materials. These materials could also increase the potency of stimulus techniques currently being used in bone regeneration. Future research would however be needed to demonstrate long term capabilities of implanted conductive and electroactive bone scaffolds.

## 6. Conclusions and Future Directions

It is easy to see how difficult it is to create a consistently functional scaffold for BTE from just one material type. This has led to the idea of using a combination of materials as well as the implementation of conductive biomaterials to ultimately create the ideal scaffolds for bone grafting. Research has been conducted using each type of material discussed; the properties present within each can be used to enhance scaffolds as well as to nullify unwanted side effects of other given materials. To eventually create the most functional scaffolds for bone tissue there has to be innovation in the process by which scaffolds are intended to function, of such, conductive materials have shown promise.

With the introduction of conductive materials being used in BTE, there is the potential for improved cellular response in addition to higher osteogenic differentiation and bone development over time. Several research studies have linked the significance of electrical conductivity to the improvement of osteogenic cell differentiation which can be exploited with conductive scaffolding materials. Tissue engineering has shown overtime to improve many aspects of biologically related impairments. Specifically, with BTE, improvements have been made, but some of the most challenging aspects of this field have been with achieving faster bone tissue development with traumatic injuries.

While bone tissue naturally exhibits piezoelectric capabilities, it has not been explored substantially within scaffold guided approaches. Conductive materials can be comprised of polymers, ceramics, and metals to make use of all positively aiding properties. In conjunction with geometric freedom, biological relevance and mechanical strengths, conductive scaffolds also provide additional biophysical and biochemical cues that can introduce a further element of bone regeneration.

Research can be done on the natural cycles of bone resorption and regeneration to get a better understanding of how osteocytes and osteoblasts function with changes in electromechanical signals. Through this, a more predictable in vivo response can be attained. While it is difficult to mimic the natural structure of bone, recent advancements in science and technology have shown the potential to attain bony scaffolds that could encourage both local and systematic biological functions in vivo. These scaffolds would only be functional with the proper selection of biomaterials, porosities that enable vascularization, and the ability to introduce and maintain necessary growth factors [203]. The future of the optimization of these properties will require an interdisciplinary approach, as there are many factors essential for bioactive bone scaffold development, which will need to be addressed through various lenses.

## Figures and Tables

**Table 1:** Advantages of synthetic and natural polymers that have been used in bone tissue engineering applications.

Type	Polymer	Advantages	Reference
Synthetic	PLA	Biodegradable; controllable geometry	[59,62]
	PCL	Biocompatibility; ease of manipulation	[67]
	PLGA	Controllable degradation	[59,68]
	PGA	Nontoxic in degradation	[62]
	PVA	Low protein absorption; high water solubility	[69]
Natural	Collagen	Naturally found in ECM; improves biocompatibility; biodegradable	[70,71]
	Fibrin	Growth factors; co-enzymes	[72]
	Gelatin	Improved osteoinduction	[73]
	Chitosan	Osteoconductivity; interaction with charged molecules; resistance to bacteria	[74,75]
	Silk	Strong natural fiber; ease of processing; controllable degradation	[76]

**Table 2:** Strengths and weaknesses of ceramics that have been used in bone tissue engineering.

Ceramic	Strengths	Weaknesses	Reference
HA	Found in natural bone tissue; biocompatible; stimulates osteoconduction	Not suitable as stand-alone supportive scaffold (often used to tune degradation)	[85,86]
TCP	High solubility; biodegradable	Low mechanical resistance; α-TCP rapid degradation	[87,88]
CaCO_3_	Flexibility in preparation; biodegradable	Reduction of compressive strength when used as additive to scaffold	[89]
BAGs	Antibacterial properties	Low fracture toughness limits implantation into load bearing bone alone	[90,91]

**Table 3:** Current conductive polymers. Reprinted from reference [145].

Name and Abbreviation	
Polypyrrole (PPy)	Poly(*p*-phenylene terephthalamide) (PPTA)
Polyaniline (PANI)	Polyacetylene (PAc)
Poly(3,4-ethylenedioxythiophene) (PEDOT)	Poly(isothianaphthene) (PITN)
Polythiophene (PTh)	Poly(*a*-naphthylamine) (PNA)
Polythiophene-vinylene (PTh-V)	Polyazulene (PAZ)
Poly(2,5-thienylenevinylene) (PTV)	Polyfuran (PFu)
Poly(3-alkylthiophene) (PAT)	Polyisoprene (PIP)
Poly(*p*-phenylene) (PPP)	Polybutadiene (PDB)
Poly(*p*-phenylene sulphide) (PPS)	Poly(3-octylthiophnene-3-methylthiophene) (POTMT)
Poly(*p*-phenylene vinylene) (PPV)	Poly(*p*-phenylene terephthalamide) (PPTA)

**Table 4:** Conductivity of bone tissue and some conductive polymers. Modified from reference [145].

Tissue/Conductive Polymer	Conductivity (S cm^−1^)
* Cancellous Bone	1.6 × 10^−3^–2.0 × 10^−3^
* Cortical Bone	5.8 × 10^−4^–6.3 × 10^−4^
Polypyrrole (PPy)	1 × 10^2^–7.5 × 10^3^
Polyaniline (PANI)	30–200
^†^ Poly(3,4-ethylenedioxythiophene) (PEDOT)	10–1 × 10^3^
Polythiophene (PTh)	10–1 × 10^3^
Poly(*p*-phenylene) (PPP)	1 × 10^2^–1 × 10^3^
Poly(*p*-phenylenevinylene) (PPV)	3–5 × 10^3^
Polyacetylene (PAc)	1 × 10^3^–1.7 × 10^5^

* Obtained from [156]; ^†^ Obtained from [157].

## Data Availability

Not applicable.
